# Engineered stromal vascular fraction for tissue regeneration

**DOI:** 10.3389/fphar.2025.1510508

**Published:** 2025-03-13

**Authors:** Jianfeng Liu, Yiwei Li, Yanan Zhang, Zhiwei Zhao, Bin Liu

**Affiliations:** ^1^ Department of Hand and Foot Surgery, Orthopedics Center, The First Hospital of Jilin University, Changchun, China; ^2^ Engineering Laboratory of Tissue Engineering Biomaterials of Jilin Province, Changchun, China

**Keywords:** wound healing, bone and cartilage regeneration, nerve regeneration, stromal vascular faction, clinical trial

## Abstract

The treatment of various tissue injuries presents significant challenges, particularly in the reconstruction of large and severe tissue defects, with conventional clinical methods often yielding suboptimal results. However, advances in engineering materials have introduced new possibilities for tissue repair. Bioactive components are commonly integrated with synthetic materials to enhance tissue reconstruction. Stromal vascular fraction (SVF), an adipose-derived cell cluster, has shown considerable potential in tissue regeneration due to its simple and efficient way of obtaining and its richness in growth factors. Therefore, this review illustrated the preparation, characterization, mechanism of action, and applications of engineered SVF in various tissue repair processes, to provide some references for the option of better methods for tissue defect reconstruction.

## 1 Introduction

Despite the human body’s regenerative and self-healing capabilities, healing takes considerable time and requires assisted treatment. Some tissue injuries cause significant problems; for instance, severe nerve defects lead to sensory and motor disabilities as neurons are nonproliferating. Tissue engineering has been put forward as an important approach for promoting tissue regeneration and achieving better prognosis. Langer and Vacanti first described the concept of tissue engineering in 1993 (Langer and Vacanti, 1993). With the integration of material chemistry, biology, and medicine, various tissue engineering materials have been fabricated for tissue repair.

One of the essential focuses of tissue engineering is seeding cells. Stem cells have confirmed their effectiveness in tissue reconstruction, especially in immune modulation, angiogenesis, paracrine activity, and even differentiation *in situ* (Bunnell, 2021). They have abundant resources such as bone marrow, adipose tissue, dental pulp, and blood. However, the process of isolation and proliferation is time-consuming, and the procedure is invasive. There are potential risks of tumorigenesis due to inappropriate disposal, and ethical conflicts also limit the use of stem cells.

**FIGURE 1:**
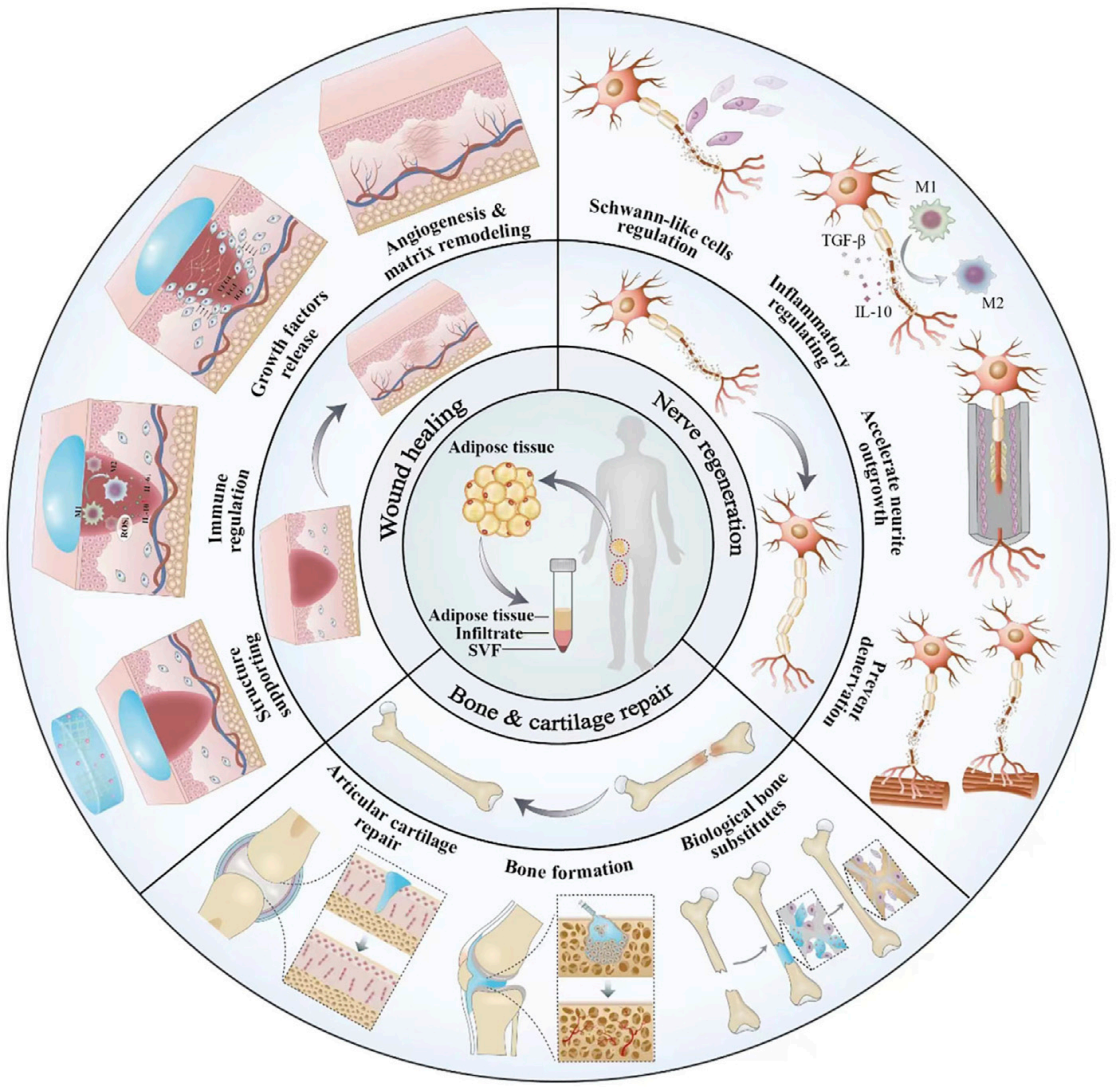
Engineered adipose tissue-derived SVF in different tissue reconstruction

Stromal vascular fraction (SVF), which is obtained from adipose tissue, has received more and more attention due to its comprehensive potential as an alternative graft material and its multiple advantages. Adipose tissue not only plays an important role in insulation and energy reservation but also acts as an immunologic barrier and endocrine tissue owing to its complex composition. In addition to adipocytes, adipose tissue contains progenitors of adipocytes, vascular endothelial cells, pericytes, immune cells, and many other cells. Various cell constituents lay the foundation for diverse tissue repair. Fat grafting is the main application of adipose tissue, mainly for special fillings. But the final fat graft retention rate was between 20% and 80% according to previous reports (Gause et al., 2014; Limido et al., 2023). SVF is obtained through emulsification, enzymic digestion and centrifugation of lipoaspirate during fat grafting, as the necessary pathway of adipose-derived stem cell (ADSC) isolation. Zuk first isolated a population of cells from human adipose tissue and verified their multilineage differentiation potential in 2001, which was named processed lipoaspirate (PLA) (Zuk et al., 2001). Adipose tissue became another source of stem cells which were generally from the embryo and bone marrow before. Interestingly, adipose tissue contains more stem cells than other mesenchymal tissues (John et al., 2006). Matsumoto described the technique of cell-assisted lipotransfer in 2006, where SVF derived from the aspirated fat was mixed with the fat again, resulting in a better survival rate of 35% on average (Matsumoto et al., 2006). SVF-loaded engineering materials for varied tissue repair have been reported in recent years, involving wound healing (Deng et al., 2017), bone and cartilage repair (Guerrero et al., 2022; Singh et al., 2022), peripheral nerve regeneration (Tuncel et al., 2015; Shimizu et al., 2018), skin rejuvenation (Suh et al., 2019; Surowiecka and Struzyna, 2022), and the repair of some special tissues, such as esophageal (Nachira et al., 2021) and hand repair (Nseir et al., 2017) (Figure 1). Due to the abundance of stem cells, minimum invasion, simple extractive process and plentiful resources, surgeons and researchers have reached a consensus that the engineered SVF is a promising and efficient option for tissue repair.

This review reveals the characterizations, mechanisms and applications of engineered SVF in wound healing, bone and cartilage reconstruction, peripheral nerve regeneration, and clinical trials.

## 2 Preparation, characterization, and application of SVF

### 2.1 Preparation of SVF

The methods for obtaining SVF reported in previous literature mainly consist of enzymatic digestion and nonenzymatic (mechanical) isolation, as well as optimized methods based on these. The methods reported in adipose harvesting are roughly similar. In brief, the adipose tissue was harvested and cut into pieces under sterile condition. In the enzymatic digestion method, the cells dissociated after 30–45 min incubated at 37°C in 0.075% of collagenase I solution. The cell suspension was then passed through the filter. After centrifugation, the pellet was collected by discarding the upper fat component and the aqueous solution. Adipose-derived stem cells were obtained by continuing the culture and passage of the resuspended pellet. Comparisons of advantages and disadvantages between SVF and ADSC were summarized in Table 1.

**TABLE 1:** Advantages and disadvantages of SVF and ADSC.

	SVF	ADSC
Advantages	No histocompatibility barriers (autografts) Abundant resources	Stable cell phenotype
	Easy isolation	Hypoimmunogenic
	High safety	Allogenic and/or autologous Abundant resources
	Multiple cellular subsets	Multifunctional
	Multifunctional	Culturally expandable
Disadvantages	Limited number of cells	Long exposure time
	Limited clinical evidence	Condition for cell culture
	Lack of standard procedure	Need for enzyme digestion
	Ethical concerns	Ethical concerns

Nonenzymatic isolation is conducted by mechanical emulsification of the adipose tissue. As the syringes were pushed and pulled back, the minced adipose tissue or lipoaspirates were physically cut repeatedly through the Luer lock (usually the mesh diameter was tens of microns), which connected between the two syringes. SVF is isolated by mechanical forces, which break the structural integrity of ECM and periadventitial structures. The product presented not a single cellular product, but rather an aggregation of cellular debris, several kinds of cells, and ECM fraction (Condé-Green et al., 2016). Various ingredients are beneficial for tissue reconstruction but will be destroyed during enzymatic digestion. However, differences between sampling sites, emulsifier types, processing time, and separation methods may hinder large-scale SVF production.

Compared to the SVF obtained by enzyme digestion, mechanical emulsion is more suitable for *in situ* tissue engineering. Based on proteomic study, Nadia et al. found that the mechanical processing of lipoaspirate promoted wound healing by upregulating early inflammation and antibacterial pathways (Sanchez-Macedo et al., 2022). Deng suggested that ECM/SVF obtained by mechanical emulsification of lipoaspirate under serum-free conditions achieved better therapeutic effects than SVF cells obtained by enzymatic procession, mainly due to its higher growth factor concentration (Deng et al., 2017). Ding found that SVF had better elasticity when comparing the rheological properties of adipose tissue derivatives obtained though different mechanical procedure (Ding et al., 2022). SVF from mechanical emulsion works like integrated cell groups rather than single cells. The interaction between cells supports their display of more advanced properties.

### 2.2 Characterization of SVF

**FIGURE 2:**
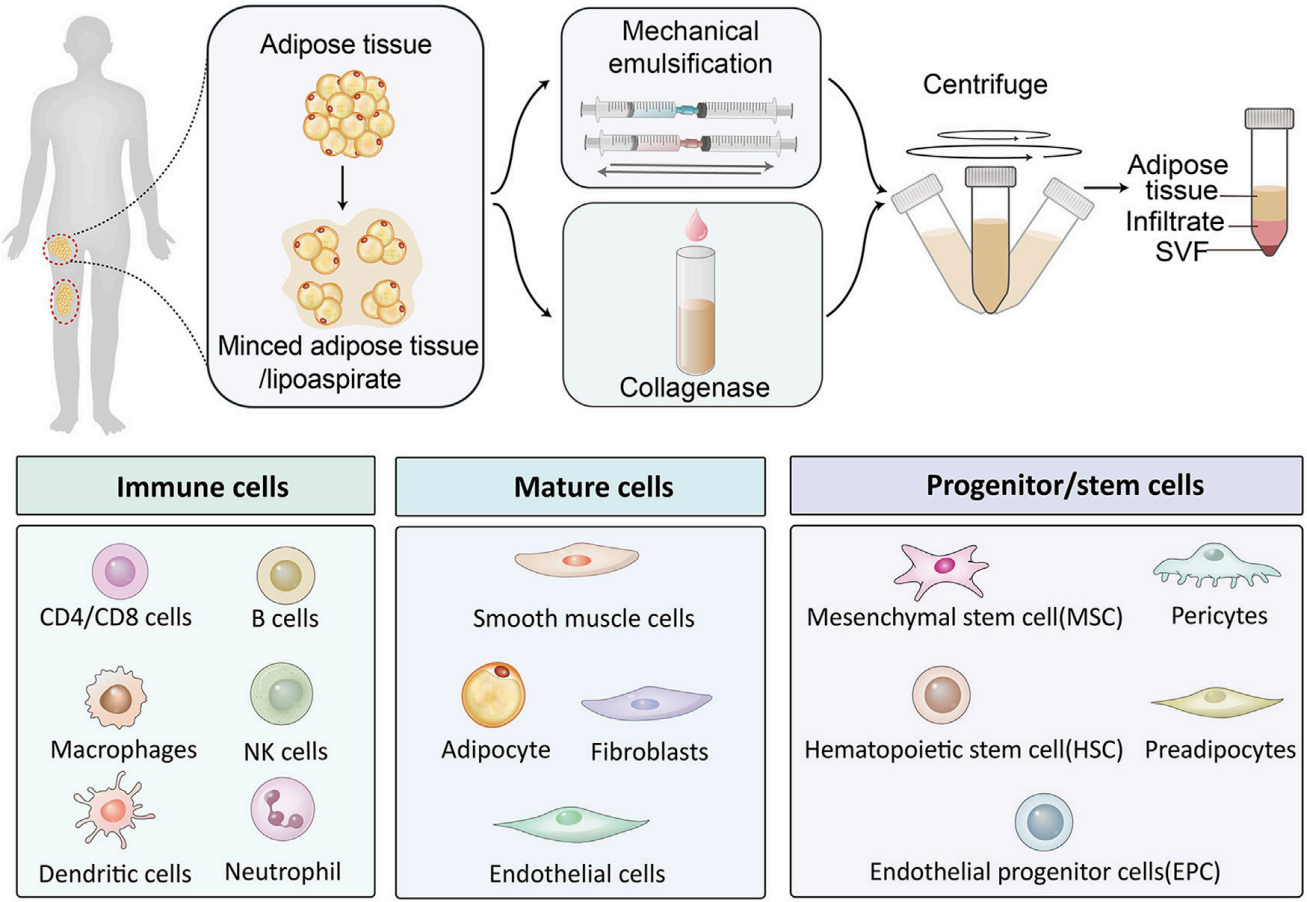
Cellular clusters in SVF are classified by function, including immune cells, mature cells, and stem/progenitor cells.

The cellular components within adipose tissue include structural cells and functional cells (Hildreth et al., 2021). Mature adipocytes occupy the majority of the adipose tissue. SVF was a cell cluster in adipose tissue after adipocytes were removed. The major cellular components in SVF are concluded as Figure 2. Cells in SVF can be divided into immune cells and non-immune cells. Specifically, immune cells include T cells (CD4^+^, CD8^+^), B cells, NK cells, dendritic cells, neutrophils, and macrophages. Non-immune cells can be classified as mature cells and progenitor cells (including stem cells) based on their differentiation state. Although the specific classification is different, the types of cells in SVF are similar in different literature. This is consistent with recent studies of single-cell sequencing of SVF-related samples (Gu et al., 2024). Interestingly, although the types of cells in SVF are consistent, the proportions of various cells measured under different conditions are significantly different, which may be related to the differences between species, sites of sampling, and processing protocols, and even healthy or not (Chun et al., 2019; Hildreth et al., 2021; Girard et al., 2022; Major et al., 2022; Gu et al., 2023; Gu et al., 2024).

The expression of surface markers is determined by the cellular components. Surface markers of CD29, CD44, CD73, CD90, and CD105 always express positive and CD45 negative due to the existence of ADSCs (Dykstra et al., 2017). The process of neovascularization was promoted via their release of growth factors such as VEGF, HGF, TGF-β et al (Chun et al., 2019) Hematopoietic and endothelial markers (CD34 and CD 31) also express positive in SVF. Nevertheless, Reis compared freshly isolated and cultured SVF for 5 and 8 days and found that in freshly isolated SVF, the ratio of CD31+ and CD34+ were higher, showing stronger angiogenic properties, but as the cells were cultured, the expression ratio decreased, showing more stem cell potential (Costa et al., 2017). The finding that the presence of CD34 on freshly isolated MSCs gradually lost with successive culture was supported by additional literature (Guo et al., 2016). Miñana found that the secretion of VEGF and bFGF by CD14+ monocytes found in SVF makes a great contribution in tissue vascularization (Navarro et al., 2014).

### 2.3 Application of engineered SVF

**FIGURE 3:**
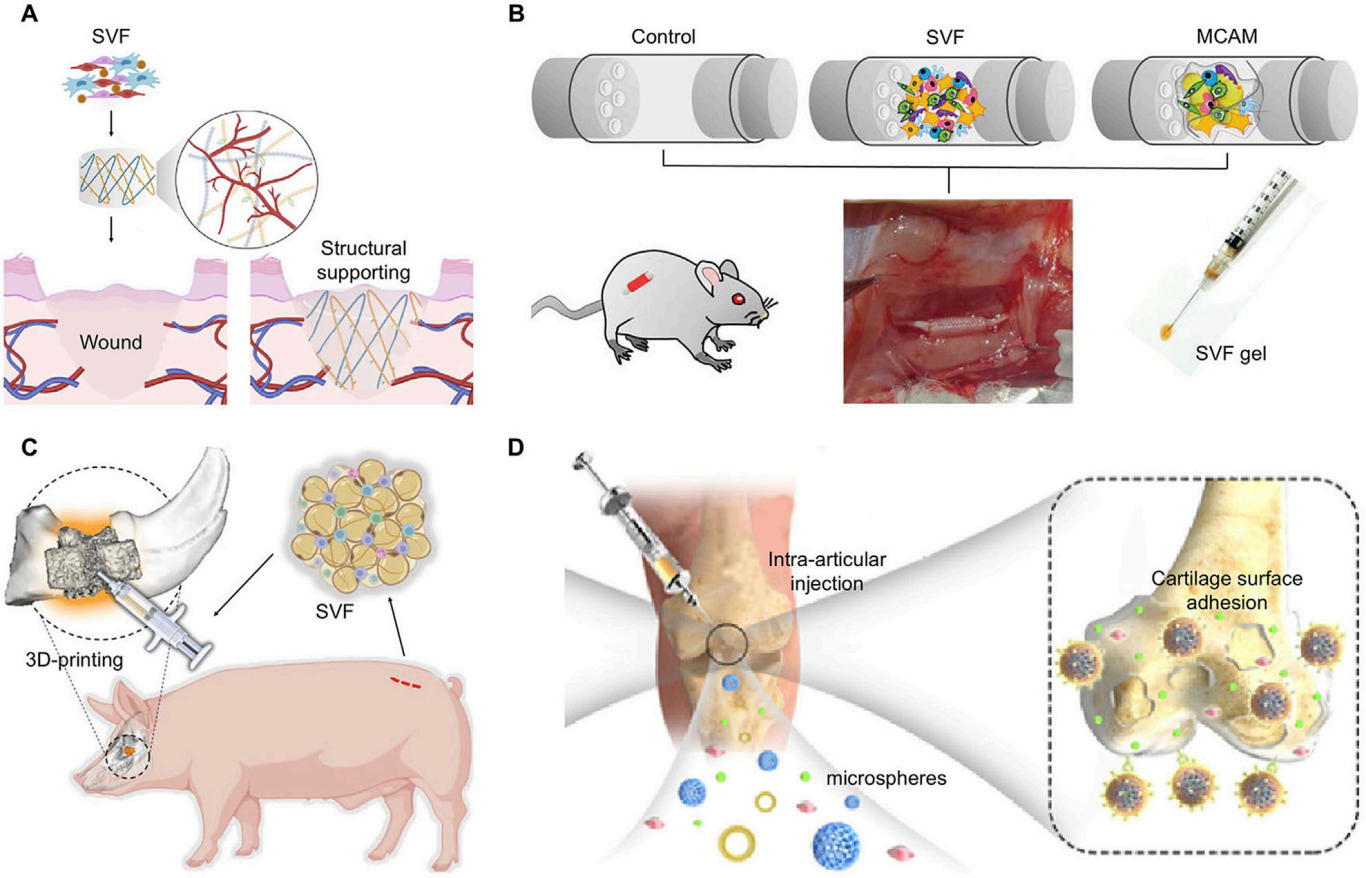
Applicable modes of engineered SVF. **(A)** SVF loaded in hydrogel for wound healing. Copyright with permission (Moreira et al., 2022) Copyright 2022 Elsevier. **(B)** SVF and MCAM filled in conduits for nerve defect regeneration. Reproduced with permission (Sawai et al., 2023) Copyright 2023 Wolters Kluwer Health, Inc. **(C)** Autologous SVF injected into a 3D-printed scaffold for bone defects. Reproduced with permission (Singh et al., 2023) Copyright 2022 Wiley. **(D)** Adipose extraction immobilized in porous microspheres for osteoarthritis. Reproduced with permission (Han et al., 2022). Copyright 2022 Elsevier.

SVF is an aggregation rich in cells and concentrated growth factors. Traditional methods of drug or growth factor delivery provide a fixed dosage. The post-delivery effect mainly depends on careful delivery design,release efficiency, and duration. Stromal vascular fraction(SVF), as a special cell population, can facilitate the transplantation of cells with secretory function to a designated site(doi:10.1186/s13287-024-03946-3.) These cells directly respond to the local microenvironment of tissue injury and maintain continuous repair functions. This dynamic and continuously adaptive autologous transplantation may be more conducive to the reconstruction of tissues. The simple extraction and injectability of SVF allows it to be used for local tissue filling soon after obtained. SVF combined with autologous fat grafting greatly improved the retention rate and reduced the need for repeated injections (Matsumoto et al., 2006). The role of injectable SVF in facial rejuvenation is not only manifested in volume filling (Schipper et al., 2022), but it still plays an important role in other aesthetic needs, such as scars reduction and hair growth (Suh et al., 2019). SVF has an effect on tissue regeneration when loaded in various materials such as gels (Figure 3A) (Moreira et al., 2022), conduits (Figure 3B) (Sawai et al., 2023), scaffolds (Figure 3C) (Singh et al., 2022), and microsphere carriers (Figure 3D) (Han et al., 2022). Engineered SVF meets the needs of tissue repair such as wound healing, bone and cartilage repair, and nerve regeneration. The biological functions of SVF help to improve the repair effect of biomaterials.

The interactions between SVF and biomaterials are varied. By the attachment of gels, scaffolds, or other biological materials with the damaged tissue, SVF stably colonized in the required region, and played roles directly, especially in complex parts such as articular cartilage (Dong et al., 2017; Li et al., 2020). The coated SVF can be more rationally distributed in the biological material, so that the SVF can not only maintain contact and interaction with the tissue cells, but also avoid the extrusion and energy consumption caused by excessive accumulation of cells (Chouhan et al., 2019). Some surface topological designs of materials regulate the biological function of cells, such as stimulating the differentiation of stem cells, to get better effects (Chaaban et al., 2023). By regulating the number and distribution of cells, it can also indirectly affect the degradation of materials, the release of internal functional groups, and guide the growth of tissues (Qian et al., 2018; Ramburrun et al., 2021).

## 3 Applications of engineered SVF for tissue regeneration

### 3.1 Engineered SVF for peripheral nerve regeneration

The course of nerve regeneration is complicated. Some important intervention nodes have attracted more attention, such as Wallerian degeneration, phagocytosis of debris, axonal extension and myelination. Multiple factors play a role in these phases. SVF has been an applicable stimulator for nerve regeneration.

#### 3.1.1 Regulation of schwann/schwann-like phenotype cells

Axon regeneration in central nervous system is difficult, in contrast, peripheral nerve exhibits highly regenerative capacity, which is due to the plasticity of Schwann cells. Phenotypic transformation of Schwann cells works on disintegrating distal axons, clearing axon and myelin debris, forming regeneration tracks, and promoting axonal regrowth (Nocera and Jacob, 2020). Based on the continuous achievements of stem cell technology, researchers have tried to explore a new way to improve the outcomes after nerve injury. They have focused on the differentiation of stem cells from different sources into Schwann/Schwann-like phenotype cells as a new strategy to accelerate nerve regeneration.

ADSCs and other progenitor cells constitute a considerable proportion of the SVF. Therefore, SVF exhibits multilineage differentiation potential. Stem cells or cell spheres coated with biological materials are converted to neural lineage-like or Schwann-like cells and express specific markers such as glial fibrillary acidic protein, glial cell line-derived neurotrophic factor, and S-100β (neural specific protein) (Huang C.-W. et al., 2020; Zhang et al., 2022). Hu et al. found that the differentiation of stem cells into SCs would be enhanced by the conductive properties of oriented nanofibers (Hu X. et al., 2020). More neurotrophic factors and other growth factors are secreted by Schwann cells differentiated from ADSCs than by undifferentiated ADSCs. The exosomes derived from the differentiated ADSCs express several miRNAs that are related to nerve regeneration and enhance the biological therapeutics effects (Liu et al., 2022). The adhesion, migration, proliferation, and cell death of Schwann/Schwann - like phenotype cells, as cell behaviors, would be regulated by co - culture with stem cells (Faroni et al., 2013; Resch et al., 2018). The axonal outgrowth of DRG neurons was not only accelerated but also orientationally guided, which prevented the formation of neurofibroma by co-culturing Schwann cells with endothelial cells on the scaffold with a modified topological morphology (Zheng et al., 2022).

#### 3.1.2 Inflammatory regulation

An early intervention to excess inflammatory response is beneficial for tissue repair. SVF plays a role in regulating the inflammatory micro-environment. Firstly, various cytokines and chemokines that modulate the activity and differentiation of immune cells are secreted by SVF. For instance, by co-culturing with splenocytes isolated from a mouse with autoimmune encephalomyelitis (EAE) mouse, IL-10 and TGF-β, promoters of regulatory T cells, were expressed considerably more in SVF than in ADSCs. Adipose-derived SVF alleviates infiltration of astrocytes and alternative activation of macrophages in the CNS (Bowles et al., 2017). A rat hind limb allograft model was performed to validate the role of adipose derived SVF in immunomodulation. SVF prolonged the graft survival, as well as decreased proliferation and infiltration of T cells and increased Treg expression (Chen J. et al., 2021). While there is a paucity of reports on the direct effects of SVF in regulating inflammation during nerve regeneration, its potential in immune response regulation suggests it could support rapid nerve reconstruction through immune regulation.

#### 3.1.3 Accelerate neurite outgrowth

For faster axonal regeneration and better nerve recovery, a collection of exosomes produced by ADSCs or directly induced Schwann-like cells have been often adopted, even though it costs much time and many procedures in cell induction culture *in vitro*, causing clinical application challenges. Neurite outgrowth is highly valued in the reconstruction of peripheral nerve defects. Excitingly, optimized engineered SVF induces impressive outcomes such as promoting axon growth, alleviating muscle atrophy and nerve regeneration (Mohammadi et al., 2014; Kappos et al., 2015; Shimizu et al., 2018). Exosomes and exocellular vesicles, isolated from SC-like phenotype cells obtained and induced from adipose tissue, have been proven to enhance neurite outgrowth (Ching et al., 2018).

#### 3.1.4 Reduce the denervated atrophy of target muscle

Muscle atrophy caused by denervation is one of the most common manifestations after nerve injury and the most direct cause of reduced or lost function. The outcome of an integrative multi-omics research revealed that oxidative stress, mitochondrial dysfunction and metabolic disorders were involved in the pathogenesis of muscle atrophy (Wang et al., 2024). Enhanced axonal regeneration and reduced muscle atrophy can be achieved by differentiating ADSCs into a Schwann-like phenotype. However, El-Habta verified that the hepatocyte growth factor is expressed and secreted by SVF at sufficient concentrations to enhance myoblast proliferation. His ensuing experiments proved that SVF directly injected into denervated target organ muscles could produce an anti-apoptotic effect and reduce muscle atrophy (El-Habta et al., 2018; El-Habta et al., 2021).

### 3.2 Engineered SVF for wound healing

Skin defects are common. They are mainly caused by trauma, but can also be secondary to wide tumor resection, scar release, radiation-induced injury (Yang et al., 2023), or diabetic ulcer. Engineered SVF has been an ideal candidate for wound healing and skin regeneration. It works through all four phases (hemostasis, inflammation, proliferation and remodeling) of wound healing via multiple mechanisms.

#### 3.2.1 Structural supporting

**FIGURE 4:**
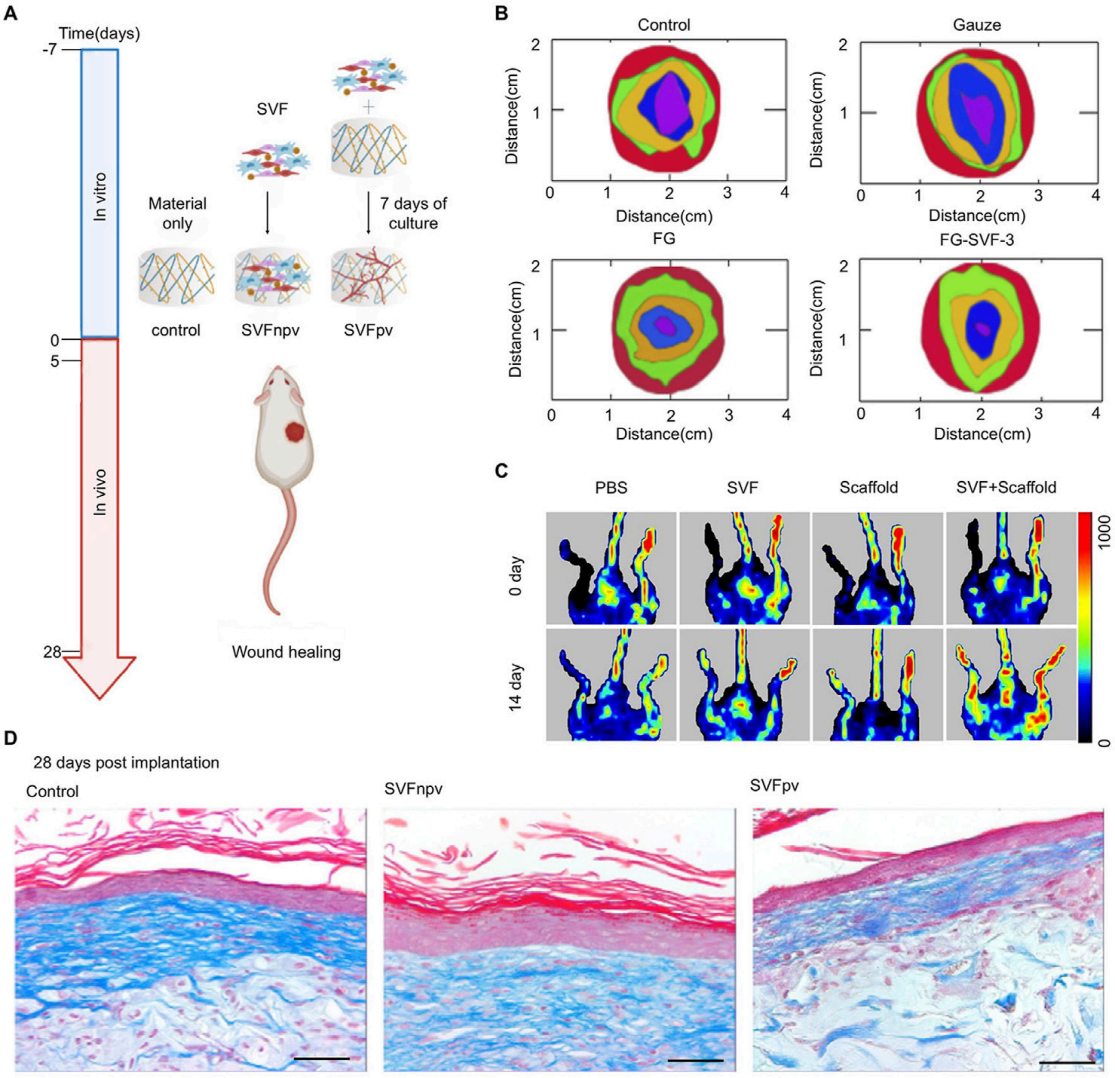
Engineered SVF for wound healing. **(A)** Schematic representation of SVF seeded in hydrogel for wound healing. Copy with permission (Moreira et al., 2022) Copyright 2022 Elsevier SVFnpv (SVF seeded in the material immediately after isolation), SVFpv (SVF cultured for 7 days *in vitro* in the material). **(B)** Wound closure with different treatments after 3, 7, 10, and 15 days. Copyright with permission Luo et al., 2024. Copyright 2024, Royal Society of Chemistry (Luo et al., 2024). **(C)** Representative laser Doppler spectroscopy images from day 0 and day 14 in a hindlimb ischemia mouse model. Copyright with permission Hu et al., 2020b. Copyright 2020 Front Bioengineering and Biotechnology (Hu et al., 2020a) **(D)** Images of Masson’s Trichrome staining at 28 days post-implantation. Scale bar = 50 μm. Copyright with permission (Moreira et al., 2022) Copyright 2022 Elsevier.

The proper proportion of components and appropriate structure design are conducive to tissue reconstruction, leading to the formation of natural tissue rather than scar tissue. For example, collagen Ⅰ, which is used as a scaffold component, may act as a raw material for skin tissue regeneration under physiological conditions during the process of degradation (Wang et al., 2023). SVF seeded in hydrogel was usually adopted in wound healing (Figure 4A). When SVF is applied alone *in vitro*, its biological activity is inhibited, and it is difficult to exert its function. Incorporating SVF with biomaterials alters its distribution, range of action, and efficiency and displays its biological effects (Luo et al., 2024). By means of 3D-culture in engineering materials, mechanical and degradational properties would be adjusted and cell behaviors would be regulated to obtain better outcomes (Dong et al., 2017). Multilayer implants fabricated by 3D-bioprinting, loaded with adipose-derived cells and matrix, supported the structure of several different cell layers, which were useful for the full-thickness repair of the skin (Zhang et al., 2023).

#### 3.2.2 Immune microenvironment modulation

Hemostasis is the first phase of wound healing, followed by the inflammatory phase. SVF acts as a regulator of the local immune microenvironment by releasing anti-inflammatory mediators or other related factors in wound healing. SVF regulates local inflammatory responses via promoting the expression of IL-10 and TGF-β (Bowles et al., 2017). TGF-β, as an important biological factor, is involved in almost the whole process of wound healing (Deng et al., 2024). Macrophage regulation is crucial in tissue regeneration. A recent single-cell sequencing study has shown that Pdpn + macrophages in adipose tissue enhanced the expression of anti-inflammatory IL-10, thereby ameliorating arterial dysfunction and inflammation in diabetic rats (Li et al., 2024).

#### 3.2.3 Rich in immediately available growth factors and cellular productions

Rapid skin regeneration relies on masses of factors. The role of various cytokines involved in the regulation of biological activities is undoubtedly of great influence. Adipose tissue can be regarded as a complex endocrine organ. Adipose-derived SVF contains a variety of growth factors. The conditioned medium of the SVF-gel obtained after mechanical emulsification exhibited higher concentrations of growth factors compared with conditioned media from untreated SVF or adipose-conditioned medium (CM) (Deng et al., 2017). A 5 - fold increase in the number of cells retained in a chronic full - thickness wound model was observed *in situ* when uncultured SVF was loaded on a nanosheet rather than seeded prior (Aoki et al., 2024). The concentration of SVF also affects the wound closure rate Figure 4B shows that the SVF ratio at 30% (w/v) in fish gelation got the fastest wound closure rate in different treatments (Luo et al., 2024).

#### 3.2.4 Angiogenesis and matrix remodeling

Angiogenesis is crucial during tissue regeneration. SVF promotes wound healing by focusing on angiogenesis and matrix remodeling (Bi et al., 2019). With the capacity to accelerate angiogenesis by reassembling endothelial cells, SVF was applied to alleviate ischemia and prevent fat absorption in fat grafts (Koh et al., 2011). In the ischemia model formed by femoral artery ligation, limb ischemia was alleviated by means of intramuscular injections of SVF and adipose-derived MSCs. They both got impressive promotions in blood flow, muscle fiber injury, and angiogenic properties compared with the control group (Kim et al., 2024). Furthermore, another similar study demonstrated that SVF seeded in a scaffold achieved better therapeutic improvement in perfusion recovery than that treated with the injection of SVF (Figure 4C) (Hu C. et al., 2020)

The interplay between SVF and the extracellular matrix (ECM) is a critical aspect of its regenerative potential. Cellular production of SVF components, particularly ECM proteins, and factors that contribute to the remodeling of the wound bed. This remodeling process involves the degradation of the provisional ECM, deposition of new ECM components, and modulation of ECM stiffness, which collectively facilitate cell migration, proliferation, and tissue reorganization. Moreira et al. explored prevascularization by culturing SVF on the scaffold for 7 days before repairing, aiming at promoting ECM deposition and improving the quality of wound healing. However, no significant advantage was presented (Figure 4D) (Moreira et al., 2022).

### 3.3 Engineered SVF for bone and cartilage repair

#### 3.3.1 Articular cartilage repair

**FIGURE 5:**
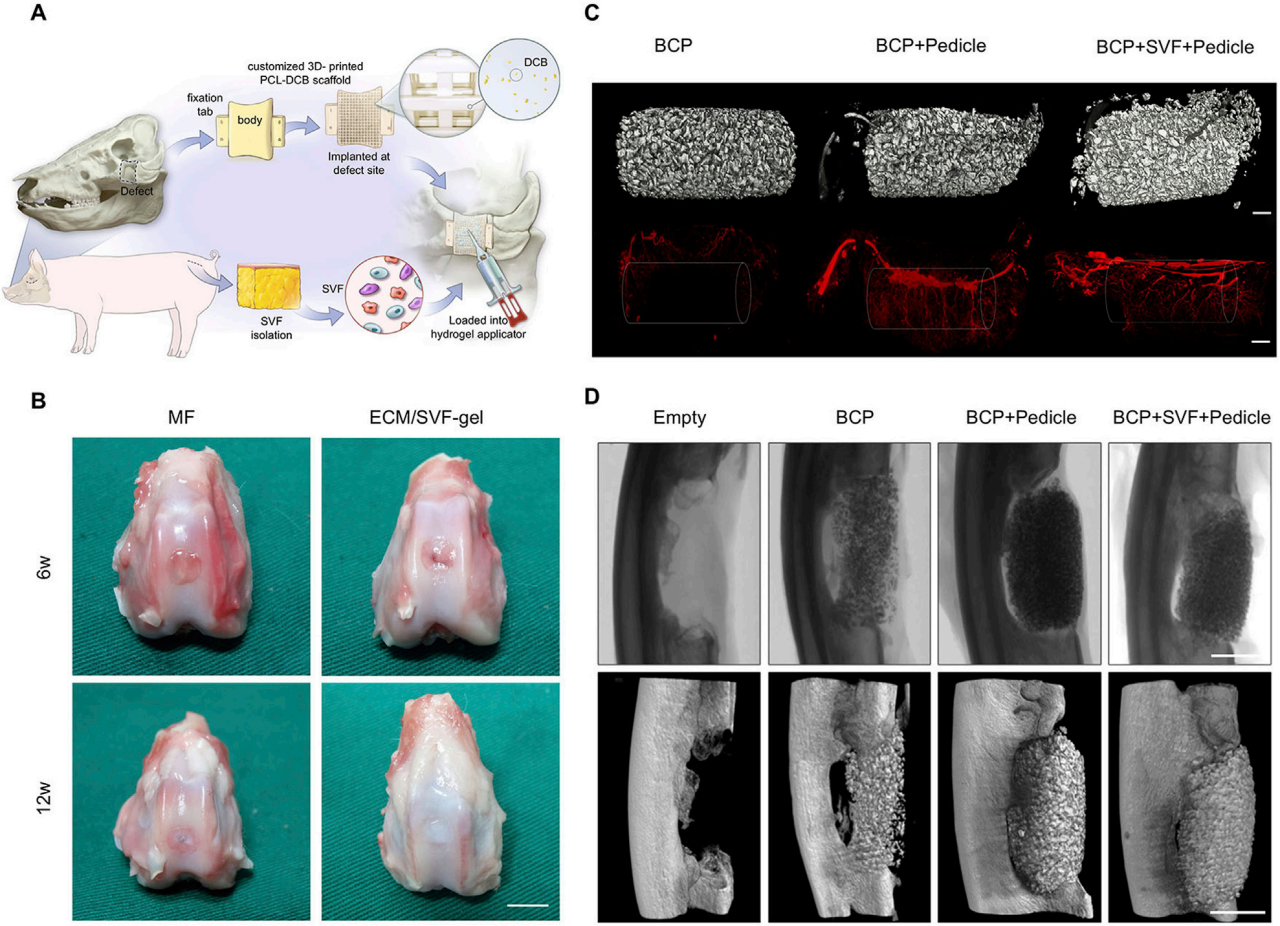
Engineered SVF for bone and cartilage reconstruction. **(A)** SVF loaded into hydrogel was injected into a 3D-printed scaffold for bone defect reconstruction. Copyright with permission (Singh et al., 2022) Copyright 2022 Elsevier. **(B)** Macroscopic evaluation at 6 weeks and 12 weeks after knee repairment. Copyright 2020 Li et al., Frontiers in Cell and Developmental Biology (Li et al., 2020). Scale bars = 5 mm. **(C)** 3D micro-CT reconstructions before (up) and after (down) chamber decalcification, which were implanted in inguinal sites for 8 weeks after a vascular contrast agent was administered. Scale bars = 1 mm. Copyright with permission (Vidal et al., 2020). Copyright 2020 Elsevier. **(D)** Micro-CT radiographs and reconstructions of ulnar defects in rabbits at 8 weeks. Scale bars = 10 mm. Copyright with permission (Vidal et al., 2020). Copyright 2020 Elsevier.

SVF was often used either alone or in combination with platelet-rich plasma to treat osteoarthritis (OA). OA is a degenerative disease of articular cartilage caused by chronic articular damage. Engineered SVF promotes cartilage regeneration, reduces the patient’s pain, and improves the treatment effectiveness of OA. The characteristics of SVF make it injectable (Figure 5A) (Singh et al., 2022). Li et al. applied ECM/SVF-gel, which is obtained by mechanical shifting and centrifugation, to articular cartilage defects and achieved notably therapeutic effects (Figure 5B) (Li et al., 2020). Fotouhi et al. reviewed the literature and came to the conclusion that autologous SVF injection is a safe and beneficent technique for treating osteoarthritis (Fotouhi et al., 2018). Chen et al. used collagenase to isolate “nanofat” and ADSCs from adipose tissue and made an intra-articular injection into a rat model of monoiodoacetate induced arthritis. The results of animal experiments and subsequent clinical retrospective studies showed pain relief and cartilage regeneration of knee arthritis and had achieved satisfactory results (Chen Z. et al., 2021). Immune regulation is an important part of the effective treatment of OA. Lee et al. derived SVF from OA patients and further isolated ADSC, and he found that high expression of IL-6 significantly enhanced immunomodulatory properties, alleviated cartilage degeneration by inhibiting RANKL and reducing the occurrence of osteoclasts (Lee et al., 2024).

#### 3.3.2 Bone formation

Some specific cytokines, polypeptides, or growth factors are often mixed to enhance the biological activity of bone-repairing materials. Bone morphogenetic protein (BMP) (Loozen et al., 2018), Biphasic Calcium Phosphate (BCP) (Park et al., 2022), Hydroxyapatite (HA) (Khan et al., 2022), and β-tricalcium phosphate (β-TCP) (Prins et al., 2016), which play crucial roles in the growth of bone and cartilage, are the most used ingredient to enhance bone healing. Hydrogels maintain a sustained release of active factors from the material. By combining SVF with injectable hydrogels, which release BMP-2 stably, Lee et al. found that the osteogenic differentiation and angiogenesis of the materials were enhanced, and the outcomes of preclinical models revealed that this combination resulted in superior spinal fusion (Lee et al., 2025). Direct addition may reduce the activity or even denature the active ingredient, while gene transfection with specific sequences was considered an ideal method and is widely used. Although MSC and SVF have the potential for osteogenic differentiation, a higher bone volume has been shown to be constructed by the delivery of BMP to several different cells compared with the control group. Thus, transfection delivery of BMP is feasible regardless of cell type (Loozen et al., 2018).

SVF is not omnipotent. The addition of various auxiliaries and optimization of conditions are required to enhance its repair effect in many tissue repairing applications. Harvestine et al. demonstrated that cell-derived ECM promoted cell retention and the osteogenic differentiation of SVF, compensating for the inferior osteogenic differentiation ability of SVF (Harvestine et al., 2018). After revascularization *in situ*, higher bone regeneration was confirmed in the synthetic bone graft, compared with the control group (Vidal et al., 2020).

#### 3.3.3 Biological bone substitutes

Large bone defects are often caused by primary or secondary tumor invasion, long segments, large area infections, and debridement after severe destructive injuries. At present, autologous vascularized fibula or iliac bone grafting is the most used method for treating large bone defects. However, the autologous sources are scarce, leaving great trauma and functional losses. Bioactive bone fillers in tissue engineering materials are urgently and widely demanded. Synthetic materials are convenient and efficient as structural fillers for bone defects, but the application of cellular productions and bioactive factors are important means to improve the biological activity of materials and accelerate bone regeneration. As an ideal active ingredient, SVF has been widely used in tissue engineering materials for bone defect repair.

The experimental results of large animal bone defect models revealed that 3D-printed scaffold combined with autologous SVF could significantly induce bone formation and achieve the integration of bone and materials (Singh et al., 2023). However, it is noteworthy that the osteoconductive bone growth by printed scaffold with the matched shape of the defect is mainly located at the periphery of the scaffold, which may result in heterotopic ossification and affect the overall repair outcome. This finding provides valuable insights for future repair strategies. The prevascularization of the scaffold *in situ* led to abundant neovascularization (Figure 5C). Rapid new bone formation within the defect was observed in the prevascularization chamber-implanted group (Figure 5D).

Engineered and inactivated hypertrophic cartilage is a promising tissue engineering material for large bone defects due to its osteoinductivity, anti-hypoxia, and strong vascularization potential. However, its inferior graft activity often leads to delayed recovery. Atanas et al. improved the repair efficiency of bone defects by engineering devitalized hypertrophic cartilage combined with SVF embedded in a fibrin gel to enhance heterotopic ossification (Todorov et al., 2016). The ability of endochondral ossification can be judged according to the maturity of hypertrophic cartilage tissue (HCT). Huang et al. mixed fractionated human adipose tissue with ceramic granules in different volume ratios, inducing HCT that formed strong, reproducible bone after heterotopic transplantation (Huang R. L. et al., 2020). A similar conclusion was drawn by Chaaban et al. who seeded SVF onto a collagen sponge to induce HCT in culture, implanted ectopic to form endochondral ossification, and found that mineralization increased with the extension of inductivity (Chaaban et al., 2023). Excitingly, Chen et al. isolated fractionated adipose tissue and recapitulated endochondral ossification in mandibular defect reconstruction in rats after differentiation into hypertrophic cartilage. The outcome was even better than that of the devitalized bone matrix, which is currently the standard bone substitute (Cheng et al., 2022).

### 3.4 Other applications of engineered SVF

#### 3.4.1 Kinetic soft tissue injury

Achilles tendinopathy is a chronic disease that seriously affects people’s life and motions. Conventional treatments, such as NSAIDs, physical therapies, and therapeutic exercises, cannot achieve satisfactory results. Although local injection of corticosteroids got a rapid improvement of pain relief, the effectiveness was transient and the risk of secondary rupture of the Achilles tendon was increased (Olafsen et al., 2018). Girolamo et al. tried to compare the application of PRP and adipose-derived SVF in the treatment of Achilles tendinopathy. After a maximum of 6 months of recovery and statistical VAS and AOFAS scores, the final conclusion was that both treatments were safe and effective, but SVF achieved the effect faster (de Girolamo et al., 2016).

The meniscus is a fibrocartilaginous disc that primarily serves to mitigate the impact forces acting upon the knee joint and protect the knee joint cartilage. During knee injuries, the meniscus is highly susceptible to damage or even tearing. Initial treatment approaches typically involve NSAIDs or physical therapeutic exercises. Meniscectomy becomes necessary when injuries are serious. What is disappointing is that the incidence of OA following partial or total meniscal resection remains a cause for concern. Rothrauff et al. seem to have found a novel and effective approach to make it possible to repair severely injured menisci. By transplanting the Stromal Vascular Fraction (SVF) into the photocrosslinked hydrogel and then applying it to a 90% full-thickness radial tear model of the medial meniscus in goats, they found that the photocrosslinked gel loaded with SVF could improve the repair of the meniscus and alleviate osteochondral degeneration (Rothrauff et al., 2019).

#### 3.4.2 Skin rejuvenation and tissue filling

Adipose tissue and its derived components have been shown to repair, regenerate and promote the recovery of surrounding tissues. In the need of skin rejuvenation or tissue filling, due to the extremely low cell retention rate of traditional fat transplantation, engineered SVF is increasingly favored by plastic and reconstructive surgeons for skin rejuvenation and tissue filling (Suh et al., 2019).

By means of microneedle injection, Verpaele et al. injected adipose-derived SVF into the skin and found that SVF regeneration ability had a more sustained and lasting effect (Verpaele et al., 2019). Cai et al. described adipose component transplantation for facial fat grafting, which achieved high patient satisfaction and without serious complications. His conclusion confirmed that SVF optimized the biological properties of adipose tissue and could be used for precise injection of facial skin rejuvenation (Cai et al., 2023).

Adipocytes and preadipocytes in SVF components make an effect on tissue filling or reconstruction, such as breast reconstruction after mastectomy. Gentile et al. concluded that SVF-enhanced fat grafting significantly improved the reconstruction contour and volume retention rate compared with the control group (63%–39%) (Gentile et al., 2012). Prosthesis implantation in combination with fat transplantation not only achieve a highly realistic appearance but also maintain an appropriate capacity. Zhao et al. reported on a 3D - printed porous prosthesis. Subsequently, adipose–derived SVF was injected into the interstices of the prosthesis, and the construct was implanted into nude mice to observe its performance. The results demonstrated that pre - implantation of adipose - tissue - integrated prostheses represents a promising approach in breast reconstruction (Zhao et al., 2023).

## 4 Clinical trials

**FIGURE 6:**
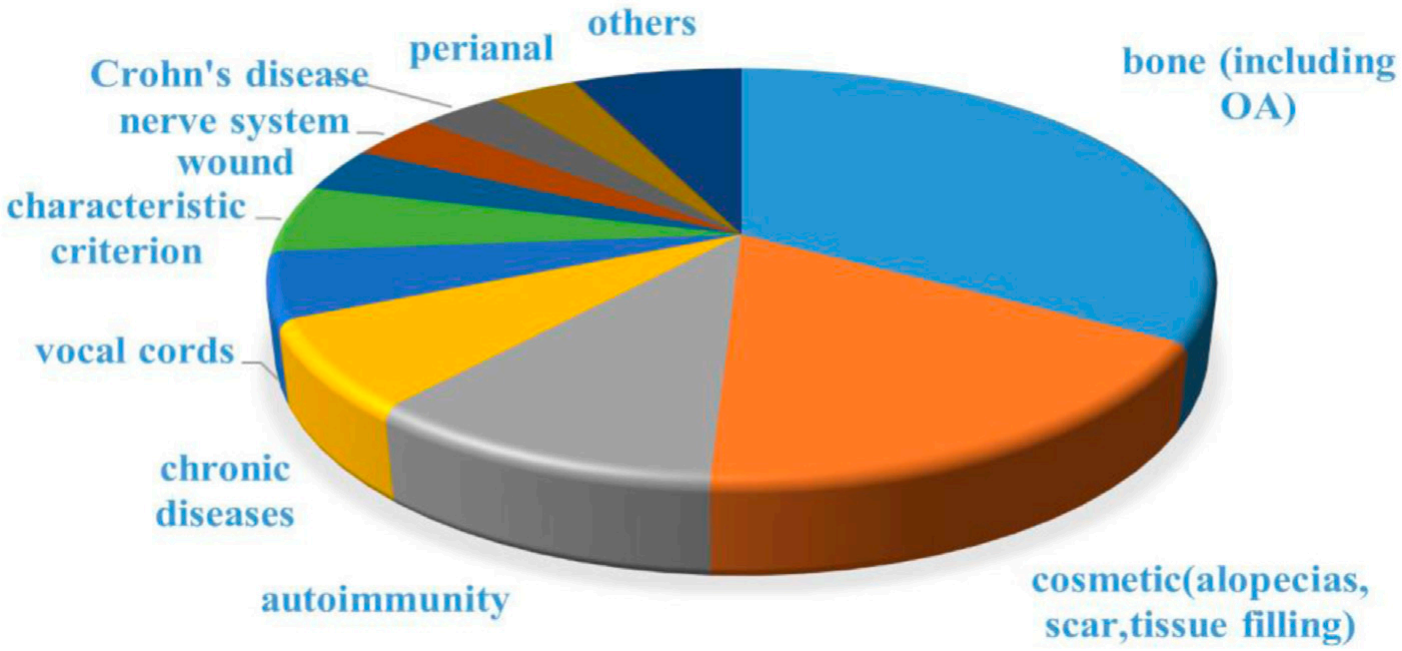
Disease distribution for clinical trials including SVF. Data was collected from clinicaltrials.gov.

The engineered adipose-derived SVF was applied to various tissues repair processes. Scholars have obtained lots of expected outcomes and published their research findings by clinical trials of SVF. The American website clinicaltrials.gov revealed that there are 57 clinical trials including SVF (10 not yet recruiting, 13 recruiting, 34 completed, and 27 with unknown status were excluded). The top three that receive the most attention from clinical researchers were osteoarthritis, cosmetic needs (including alopecia, scar control, and breast reconstruction), and autoimmune diseases (Figure 6).

SVF has demonstrated improvements in a variety of clinical trials involving immune disease regulation, scar control, wound healing et al. (Granel et al., 2015; Mattei et al., 2020; Tan et al., 2021; van Dongen et al., 2022; Dong et al., 2023; Roohaninasab et al., 2023; Wu et al., 2023; Liu et al., 2024). These results hold great promise and suggest its potential significance in many medical fields. Nevertheless, some clinical trials have verified the different conclusion that there is no significant difference between SVF and the current implemented intervention after 12 months (Mautner et al., 2023). For now, the published clinical trials reveal that the clinical application of adipose-derived SVF is safe; However, the outcomes remain controversial and data on the clinical trials are limited, although many of the conclusions to be infusive. Consequently, much more standard clinical trials from diverse clinical centers are urgently required to fully assess the effectiveness of SVF-based treatment.

## 5 Challenges and perspectives

Engineered SVF has exhibited impressive potential in different tissue reconstruction according to numerous experimental conclusions. For human beings, this approach is becoming increasingly feasible due to the abundant autograft resources, simpler processes, and shorter time required. However, what is easily neglected is that excessive harvesting of adipose tissue is extremely harmful to the human body. There are still challenges and restrictions that limit the use of engineered SVF. First, a standard procedure, evaluation criteria and good manufacturing practices are urgently needed. The format of SVF carrier should be well designed and safety must be concerned. Finally, more clinical trial data from different research centers are needed.

In summary, engineered SVF exhibits enormous potential in tissue repair. With its rich cellular composition, including a high proportion of stem cells, SVF plays an important role in multilineage differentiation, immune modulation, paracrine activity, and angiogenesis, which are essential for tissue regeneration. The engineered SVF has procured impressive progress in tissue repair, especially in severe tissue defect reconstructions. Although there are still some challenges and restrictions, the engineered SVF will do more in tissue regeneration in the future. More tissue damage to be cured by engineered SVF is expected.
